# Pre-peritoneal Fat as a Guide to Extended View Total Extraperitoneal (eTEP) Repair for Inguinal Hernia

**DOI:** 10.7759/cureus.52327

**Published:** 2024-01-15

**Authors:** Sanjay Gupta, Ashok K Attri, Zahid Iqbal Mir, Ishan Bansal

**Affiliations:** 1 Surgery, Government Medical College & Hospital, Chandigarh, IND; 2 General Surgery, Government Medical College & Hospital, Chandigarh, IND

**Keywords:** laparoscopic, preperitoneal, extraperitoneal, hernia, inguinal

## Abstract

In the extra-peritoneal approach for inguinal hernias, pre-peritoneal space creation is one of the most crucial steps. In the absence of well-defined landmarks, it is difficult to identify the correct plane of dissection, and blind dissection can sometimes lead to peritoneal injury, resulting in loss of working space. In this article, we describe our technique of pre-peritoneal space creation by following the fatty tissue of the median umbilical ligament and fatty tissue along the rectus muscle. The data of all patients (total 84) who underwent surgery with this technique between January 2021 and May 2023 were retrieved and assessed for demographics, hernia type, and perioperative complications. Except for two peritoneal injuries, there were no other intraoperative complications.

## Introduction

Of all laparoscopic/endoscopic techniques for inguinal hernia repair, total extraperitoneal repair (TEP) is still preferred because it does not involve entry into the peritoneal cavity [1]. However, the major disadvantage of this technique is limited dissection space, which can further be compromised in the case of pneumoperitoneum. To overcome this limitation, Des introduced the concept of “extended view” TEP (eTEP), based on the principle that pre-peritoneal space can be accessed from anywhere in the abdominal wall. Higher port placement in eTEP provides larger working space and better tolerance to inadvertent pneumoperitoneum [2]. However, blind dissection of the pre-peritoneal space with the help of a balloon or telescope increases the risk of peritoneal injury and bleeding, making it difficult to identify anatomical structures and dissect them further [2,3].

We observed that eTEP not only provides a large surgical field but also obviates the need for blind dissection of the pre-peritoneal space, as there is a well-defined plane of dissection that can easily be followed by sharp dissection. The fatty tissue along the rectus muscle (lateral fat pad) and the median umbilical ligament (medial fat pad) acts as a guide to identify this plane. In this article, we describe our experience with this technique of pre-peritoneal space creation for inguinal hernia repair.

## Technical report

This study included all patients who underwent surgery using the described technique between January 2021 and May 2023. It is important to note, we specifically prefer transabdominal pre-peritoneal (TAPP) repair for recurrent hernias. As a result, this study included only patients with primary inguinal hernias. The collected data encompassed various factors, including patient demographics, hernia type, operative findings, postoperative complications, and duration of hospital stay.

Surgical technique

All patients included in the study underwent surgery under general anesthesia, and catheterization was performed prior to the commencement of the procedure.

Insertion of Camera Port

For the camera port, a 10-12 mm skin incision was made, positioned 3-4 cm above the umbilicus level and medial to the linea semilunaris, on the side opposite to the hernia or larger hernia in the case of bilateral hernias. The incision was deepened to the level of the anterior rectus sheath, which was then incised to expose the rectus muscle. Using two “S”-shaped retractors, the fibers of the rectus muscle were split, and both retractors were lifted to reveal the posterior rectus sheath. A 10-mm trocar was inserted through this opening and secured to the skin with a suture to prevent displacement during the procedure. A 10-mm telescope was introduced through the trocar, and entry into the retrorectus space was confirmed by visualizing the posterior rectus sheath. Prior to dissection, carbon dioxide (CO2) was released into the extraperitoneal space for a few seconds to aid in identifying the avascular plane.

Insertion of First Working Port

To create space for the first working port, the telescope was used to perform an initial dissection up to the arcuate line. The telescope was moved laterally in a back-and-forth motion to create the necessary space. Care was taken to ensure that no tissue was left on the posterior rectus sheath during the dissection. Afterward, a 5-mm working port was inserted at the level of the arcuate line, and further dissection was conducted under vision using a mono-polar L-hook.

Dissection of Medial Space

**Figure 1:**
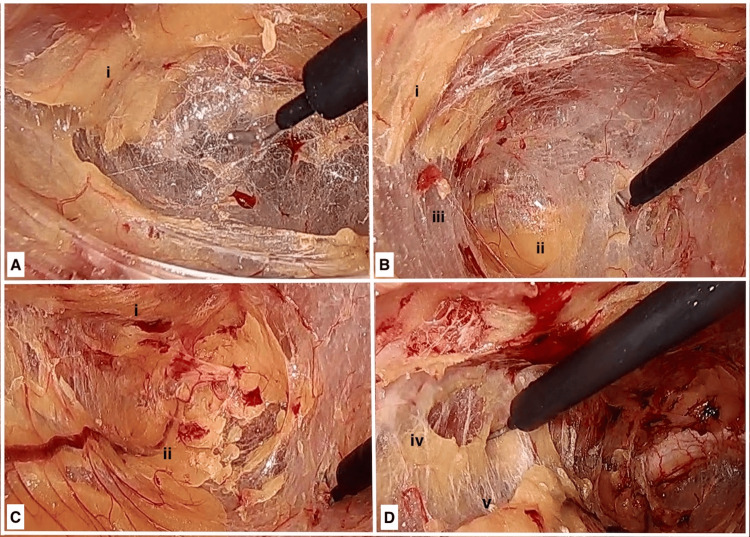
Plane of dissection to create pre-peritoneal space for left-sided hernia. (A) Below lateral fat opposite to side of hernia. (B) Between lateral fat and central fat on same side. (C) Above central fat. (D) Between central fat and lateral fat on the side of hernia. (i) Lateral fat, (ii) central fat, (iii) plane between lateral and central fat on the side of hernia, (iv) lateral fat on the side of hernia, and (v) plane between central fat and lateral fat on the side of hernia.

The fibrofatty tissue along the edge of the arcuate line was incised, allowing for identification of the avascular plane located below the “lateral fat pad,” which refers to the fatty tissue along the rectus muscle Figure 1A). The dissection continued caudally and medially toward the pubic symphysis. Medially, the dissection plane was positioned above the “central fat pad,” which corresponds to the fatty tissue of the median umbilical ligament. The presence of a vein running over the central fat pad facilitated its easy identification. Notably, a clear demarcation between the “lateral fat pad” and “central fat pad” was observed and could be readily identified through careful and precise dissection Figure 1B. The central fat pad was mobilized, and the plane between the central fat pad and the lateral fat pad on the opposite side was identified and followed laterally until reaching the level of the inferior epigastric vessels (Figures 1C, 1D).

**Figure 2:**
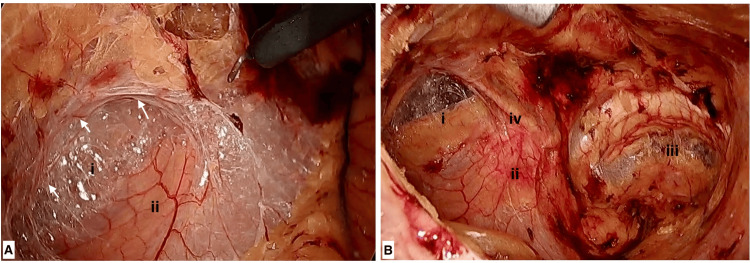
(A) Opening of Bogros space. (B) Completed dissection. (i) Bogros space, (ii) hernial sac and cord structures, (iii) Retzius’ space, (iv) inferior epigastric vessels. White arrows - Margin of the pre-peritoneal fascia

In order to access the hernial sac, any tough tissue, specifically the layer of pre-peritoneal fascia, covering the sac was dissected. This step also created a connection between the Retzius' space and Bogros' space near the origin of the inferior epigastric vessels (Figures 2A, 2B).

**Figure 3:**
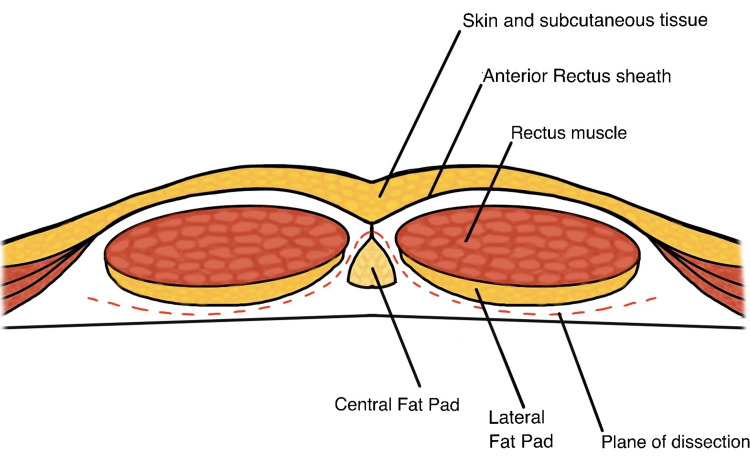
Line diagram showing the plane of dissection. Original work of authors

A line diagram illustrating the plane to be followed for the creation of the pre-peritoneal space is depicted in Figure 3.

Insertion of Second Working Port

To crossover to the opposite side above the arcuate line, the plane above the “central fat pad” was followed upwards, and the posterior rectus sheath was dissected medially. In certain cases, fibrous bands were encountered instead of a well-defined arcuate line. The second working port was inserted below the umbilicus to facilitate the procedure.

Dissection of Lateral Space and Mesh Placement

The communication between the Retzius space and Bogros space was enlarged by dividing the reflection of the pre-peritoneal fascia on the abdominal wall. This allowed for easy identification of the contents of the direct hernia entering the fascia transversalis defect or the indirect hernia sac and cord structures. Subsequently, further dissection was carried out to reduce the contents/hernial sac and parietalize the cord. A critical view of the myopectineal orifice was achieved before the placement of the mesh. The minimally edited video (edited using DaVinci Resolve 18.6) of pre-peritoneal space creation can be accessed at https://youtu.be/shJVvdWkU4U?feature=shared.

**Video 1:** Pre-peritoneal space creation for inguinal hernia

For bilateral inguinal hernias, an additional working port was inserted on the opposite side at the level of the umbilicus, while the camera port remained unchanged.

Results

Between January 2021 and May 2023, a total of 84 patients underwent surgery using the technique of pre-peritoneal space creation. The majority of patients did not experience any major complications during the initial space creation, with only two cases resulting in inadvertent peritoneal rent. A detailed breakdown of patient demographics, hernia types, and perioperative complications can be found in Table 1.

**Table 1:** Patient characteristics

Total Patients	84	
Sex	Male	78
	Female	06
Age (mean) (range)	48.8 (24-69) years	
Hernia characteristics		
Unilateral	72	
Bilateral	12	
Direct (Medial)	50	
Indirect (Lateral)	46	
Operative details		
Operative time (mean)(range)	69.3 (40-110) min	
Operative time for medial dissection (mean)(range)	17.7 (16-30) min	
Peritoneal injury	02	
Bleeding /other complication	Nil	

## Discussion

Although the extraperitoneal approach is preferred for inguinal hernia repair, its widespread adoption is hindered by the long learning curve associated with it. The procedure becomes challenging due to the absence of a well-defined space, the presence of vital structures in the vicinity, and the wide variation in the anatomy of different fascial layers [4]. Creating an adequate pre-peritoneal space is crucial for proper mesh placement and visualization of the structures involved. The primary limitation in mastering the extraperitoneal approach lies in developing proficiency in creating the pre-peritoneal space in the correct plane.

Performing blind dissection without following a proper plane, using a telescope or balloon, can result in peritoneal injury and loss of the working space. This can prolong the operative time and increase the likelihood of conversion to open surgery or alternative minimally invasive procedures [3]. Additionally, blind dissection can lead to bleeding from small vessels or the inferior epigastric artery, which further complicates the dissection and increases the risk of injury to vital structures [5]. Although various techniques have been proposed to create the pre-peritoneal space, there is no definitive landmark to guide the dissection [6,7]. The identification of fascial layers can be challenging due to their inconsistent nature, with some patients having well-defined fasciae while in others these may be thin or absent [4].

In our surgical unit, we have been performing inguinal hernia repair using an extraperitoneal approach since 2017. Initially, we used the standard totally extraperitoneal repair (TEP) technique with blind dissection using a telescope to create the pre-peritoneal space. Although the procedure was generally successful, there was always a concern about losing the correct plane and causing peritoneal injury during blind dissection. Over the course of four years, we performed TEP for inguinal hernias on 93 patients, with an average operating time of approximately 90 minutes. Eight patients experienced inadvertent peritoneal injury due to loss of correct plane of dissection when telescope alone was used for pre-peritoneal space creation below the arcuate line, with five cases requiring conversion to open surgery due to the inability to maintain the working space. In other cases, the peritoneal rent was successfully closed during the procedure using endoloop or intracorporeal suturing using absorbable suture. To address these challenges, we transitioned to the extended view TEP (eTEP) technique, which provides a broader field of view. We began creating the pre-peritoneal space using sharp dissection along the avascular plane. Blind dissection was limited to the arcuate line, as there is no risk of peritoneal injury up to this point due to the presence of the posterior rectus sheath. Through our experience, we discovered that the avascular plane could be easily identified by following the lateral and medial fat tissues. These fatty tissues consistently exist in all patients, and the higher port placement in eTEP facilitates the identification of the avascular plane by following these fat tissues. Our observations align with a recent cadaveric study by Urena et al., which reported a “trident” distribution of pre-peritoneal fat and proposed that the distribution of pre-peritoneal fat can serve as a useful guide for creating space for mesh placement. We believe that the central fat pad we follow for space creation corresponds to the middle prong of the “trident” pre-peritoneal fat [8].

In our experience with 84 cases over the last three years, we successfully identified the avascular plane in all cases except for two instances of peritoneal injury. One occurred during space creation, and the other during the parietalization of cord structures. However, both injuries were effectively closed using an endoloop. While initially, this technique may require some additional time for the surgeon to become familiar with the concept, once mastered, the operative time is significantly reduced.

## Conclusions

To conclude, we believe that creation of pre peritoneal space by following lateral and medial fatty tissue is not only safe but is also easy to learn. In the absence of standardized technique of extra-peritoneal space creation, our technique can be considered as a step forward to further standardize technique of extraperitoneal approach.
